# Phospholipase C From *Alternaria alternata* Is Induced by Physiochemical Cues on the Pear Fruit Surface That Dictate Infection Structure Differentiation and Pathogenicity

**DOI:** 10.3389/fmicb.2020.01279

**Published:** 2020-06-30

**Authors:** Yi Huang, Yongcai Li, Dongmei Li, Yang Bi, Dov B. Prusky, Yupeng Dong, Tiaolan Wang, Miao Zhang, Xuemei Zhang, Yongxiang Liu

**Affiliations:** ^1^ College of Food Science and Engineering, Gansu Agricultural University, Lanzhou, China; ^2^ Institute of Postharvest and Food Sciences, The Volcani Center, Agricultural Research Organization, Rishon LeZion, Israel

**Keywords:** *Alternaria alternata*, phospholipase C, pear fruit wax, hydrophobicity, calcium signal pathway

## Abstract

To investigate the mechanisms of phospholipase C (PLC)-mediated calcium (Ca^2+^) signaling in *Alternaria alternata*, the regulatory roles of PLC were elucidated using neomycin, a specific inhibitor of PLC activity. Three isotypes of PLC designated *AaPLC1*, *AaPLC2*, and *AaPLC3* were identified in *A. alternata* through genome sequencing. qRT-PCR analysis showed that fruit wax extracts significantly upregulated the expression of all three PLC genes *in vitro*. Pharmacological experiments showed that neomycin treatment led to a dose-dependent reduction in spore germination and appressorium formation in *A. alternata*. Appressorium formation was stimulated on hydrophobic and pear wax-coated surfaces but was significantly inhibited by neomycin treatment. The appressorium formation rates of neomycin treated *A. alternata* on hydrophobic and wax-coated surfaces decreased by 86.6 and 47.4%, respectively. After 4 h of treatment, exogenous CaCl_2_ could partially reverse the effects of neomycin treatment. Neomycin also affected mycotoxin production in alternariol (AOH), alternariol monomethyl ether (AME), altenuene (ALT), and tentoxin (TEN), with exogenous Ca^2+^ partially reversing these effects. These results suggest that PLC is required for the growth, infection structure differentiation, and secondary metabolism of *A. alternata* in response to physiochemical signals on the pear fruit surface.

## Introduction

*Alternaria alternata* is a phytopathogen that infects an array of plants, leading to the spoilage of fruits and vegetables post-harvest and during transport. The plants afflicted include pear (Tanahashi et al., 2016), peach (Iwamoto et al., 2019), apple (Gur et al., 2017), and other agricultural products (Yan et al., 2014; Kumar et al., 2018), resulting in quality degradation and large economic losses. In addition, several *Alternaria* species can produce toxic secondary metabolites including alternariol (AOH), alternariol monomethyl ether (AME), altenuene (ALT), and tentoxin (TEN; Ostry, 2008), some of which are phytotoxins that mediate fungal pathogenicity, with others defined as mycotoxins that elicit adverse effects in humans and animals (EFSA, 2011; Meena et al., 2017). Synthetic fungicides are the most commonly used treatment to combat post-harvest disease (Estiarte et al., 2017). Unfortunately, the persistent application of fungicides has gradually led to the emergence of fungicide-resistant strains in addition to environmental contamination (Yuan et al., 2019). One of the most potent counter-strategies is the development of target site-specific chemicals that inhibit fungal infections. Spore germination and attachment are critical events in the lifecycle of all fungi and represent important targets for disease control (Gupta et al., 1998). Exploring the molecular aspects of pathogen-fruit interactions has biological and economic significance as a means to develop rational alternatives for disease control.

As a latent infectious disease, *A. alternata* infection requires cellular morphogenesis, initiated by spore adhesion to the host surface, spore germination, germ tube elongation, appressorium formation, and penetration by an infectious peg that invades fruit through a stoma or epidermal wound (Li et al., 2007). Surface recognition and penetration are the most critical infection processes for plant pathogens, in which the surface sensing of hydrophobicity, hardness, and chemical composition has been implicated (Tucker and Talbot, 2001). Cuticle hardness (Kim et al., 1998; Liu et al., 2007), chemical components (Hansjakob et al., 2011), and hydrophobicity (Mendoza et al., 2009; Lanver et al., 2014) can induce spore germination and the formation of infection structures of *Magnaporthe oryzae*, *Colletotrichum trifolii*, *Botrytis cinerea*, and other pathogenic fungi. Hydrophobicity can also disrupt the dormancy of *Colletotrichum graminicola* spores and enhance spore adhesion and germination (Chaky et al., 2001). The attachment of spores to hydrophilic surfaces is relatively weak compared to hydrophobic surfaces (Mercure et al., 1994). In addition, different components of the surface wax participate in plant-pathogen interactions, inducing pathogen development (Podila et al., 1993; Tsuba et al., 2002). Emerging evidence suggests that differentiation-inducing signal components are present in wheat leaf epicuticular wax. Among them, C26-aldehyde can actively induce appressorium differentiation in *Blumeria graminis in vitro* (Tsuba et al., 2002), while C28 aldehyde octacosanal mediates host-plant recognition and infection structure differentiation in wheat stem rust fungus (Reisige et al., 2006). Other plant pathogenic fungi recognize primary alcohols to regulate infection related morphogenesis (Liu et al., 2011). Our previous study showed that the chemical composition and hydrophobicity of pear fruit cuticular wax is essential for fungal invasion through its regulation of the growth and differentiation of *A. alternata* during the pre-penetration phase (Tang et al., 2017). This highlights the role of hydrophobicity and wax as epidermal signals in pathogen-plant interactions.

Plant fungal development and infection structure differentiation in response to environmental stimuli are in-part, mediated through second messenger pathways (Uhm et al., 2003). The interaction of these pathways provides a plausible mechanism through which physiological processes can be regulated and coordinated to ensure the appropriate modification of cell growth and differentiation (Lee and Dean, 1993). Cellular signaling systems related to those that regulate the infection and morphogenesis of plant pathogenic fungi have been identified, including a heterotrimeric guanosine triphosphate GTP-binding protein (G-protein; Yamagishi et al., 2006), second messengers including cyclic nucleotides (Zhu et al., 2017), Ca^2+^ (Uhm et al., 2003), and mitogen-activated protein kinase (MAP kinase; Fang et al., 2018). Cross-talk often exists between signaling pathways that control the development and growth of pathogenic fungi, and the complexity of these interactions are dependent on the microorganism or environmental stimuli encountered (Yamauchi et al., 2004; Tsai et al., 2012; Mónica et al., 2019). Wang et al. (2009) demonstrated that the cytoplasmic cAMP levels controlled by G-proteins are key to conidial formation and the subsequent pathogenicity by *A. alternata*. The disruption of MAP kinase signaling could abolish appressorium formation, reducing disease progression in *A. alternata* (Lin et al., 2010; Lin and Chung, 2010). However, studies on the signal-mediated mechanisms of *A. alternata* during the recognition of epidermal physiochemical signals to form infection structures are less well-characterized.

Ca^2+^-signaling occurs in response to transient changes in cytosolic free Ca^2+^ concentrations in eukaryotes (Dodd, 2010). Cytosolic Ca^2+^ regulates cell signaling and a wide range of associated physiological functions, including cell development (Berridge et al., 2000; Cui and Kaandorp, 2006). Numerous pharmacological studies have confirmed the requirement for Ca^2+^ in the infectious structures of fungi including *Magnaporthe grisea* (Kim et al., 2010), *Cochliobolus miyabeanus* (Ahn and Suh, 2007), and *Colletotrichum gloeosporioides* (Kim et al., 1998). In addition, the targeted disruption of key components of Ca^2+^ signaling pathways, including calmodulin (CaM; Warwar et al., 2000) and calmodulin-dependent protein kinase (CaMK; Liu et al., 2010), leads to delayed germination and appressorium formation in a variety of plant pathogens, reducing their infectivity. Ca^2+^ signaling is initiated by environmental cues that lead to conformational changes in G-proteins. The G-proteins then activate phospholipase C (PLC), which catalyzes the hydrolysis of phosphatidylinositol 4,5-bisphosphate (PIP2) to inositol 1,4,5-triphosphate (IP_3_) and diacylglycerol (DAG). The main function of PLC is to generate IP_3_ which activates Ca^2+^ channels (Lew et al., 2015; Singh et al., 2015). In many fungi, PLC mediates various aspects of fungal development, including conidium and appressorium formation, hyphal extension and branching, and fungal pathogenicity (Oh et al., 2012; Zhu et al., 2015; Barman et al., 2018). A wide range of pharmacological agents have been used to disrupt Ca^2+^ influxes or to interfere with Ca^2+^-binding proteins. Among them, the PLC inhibitor neomycin was used to highlight the role of PLC activation during appressorium formation for *M. grisea* (Lee and Lee, 1998), *C. miyabeanus* (Ahn and Suh, 2007), and *C. gloeosporioides* (Uhm et al., 2003). PLC plays an important role in vegetative growth, conidia, Ca^2+^ homeostasis, and the pathogenicity of citrus *A. alternata* (Tsai and Chung, 2014). These results suggest that PLC regulates the morphogenesis of pathogenic fungi. Our previous studies revealed a positive correlation between pear cuticular wax hydrophobicity and appressorium formation in *A. alternata* (Tang et al., 2017). Whether PLC-mediated Ca^2+^ signaling involves this induction process requires further elucidation.

The aim of this study was to evaluate the effects of PLC-mediated Ca^2+^ signaling on spore germination and appressorium formation in *A. alternata* in response to hydrophobic and wax containing surfaces. Neomycin was used to characterize the role of PLC in *A. alternata*, a causal agent of pear black spot. PLC expression during *A. alternata* development was assessed. The regulatory role of PLC on virulence and mycotoxin production in *A. alternata* was also investigated.

## Materials and Methods

### Fungal Isolates and Culture Conditions

*A. alternata* was isolated from diseased pears obtained from the Tiaoshan Farm in Jingtai County, Gansu Province, China. Conidia were harvested from 5-day-old cultures which were grown on potato dextrose agar (PDA) at 28°C in a dark incubator. Conidial suspensions were filtered through four layers of cheesecloth to separate hyphal fragments, which were adjusted to a concentration of 10^6^ conidia/ml. Spore concentrations were determined using a hemocytometer for *in vitro* and *in vivo* assessments.

### Chemicals and Reagents

Neomycin was obtained from Beijing J&K Scientific (Beijing, China). Gelbond PAG film was purchased from Shanghai Univ-bio (Shanghai, China). Certified standards of *Alternaria* toxins, namely AME, AOH, ALT, and TEN were purchased from Shanghai Yuanye Bio-Technology Co., Ltd. (Shanghai, China). All solvents and chemicals were of analytical grade. Chemicals were dissolved in water or appropriate solvents for the production of stock solutions.

### Sampling of Cuticular Waxes

Cuticular wax was extracted according to previous methods (Tang et al., 2017). Briefly, pears were immersed, agitated twice for 30 s in 600 ml chloroform at room temperature (25 ± 2°C), washed with tap water, and dried. The solvent was filtered and evaporated at 40°C. Waxy extracts were stored in a refrigerator at 4°C for further experiments.

### Contact Angle Measurements

According to Reisige et al. (2006), hydrophobicity was evaluated through measurements of the contact angle, measured using a Drop Shape Analyzer 100 (Kruss Company, Hamburg, Germany). A larger contact angle indicated a more hydrophobic surface. Three independent measurements per surface were performed.

### Identification and Cloning of the *AaPLC* Gene Family

Members of the *AaPLC* gene family were identified through a BLAST search of the *Alternaria* (txid 5599) genomic database using PI-PLC proteins of *M. oryzae* and *Neurospora crassa*. The results indicated that *A. alternata* has three putative PLC-encoding genes. The putative *AaPLC* genes were designated *AaPLC1* (XP_018383411), *AaPLC2* (XP_018381337), and *AaPLC3* (XP_018388596). Mycelia were obtained from 5-day-old PDA cultures. Total RNA was extracted from *A. alternata* mycelia using TRNzol reagent (QIAGEN, Shanghai, China). The synthesis of cDNA was performed according to the manufacturer’s protocol. cDNA segments encoding PLC were amplified with specific primers (Table 2). Gel recovered products were ligated with a pTOPO-Blunt vector (Aidlab, Beijing, China) and transformed into the competent cell (TransGen, Beijing, China), inserted from a positive clone excised to yield plasmid *pAaPLC*. Samples were sequenced and analyzed.

**Table 1:** Hydrophobicity of the Gelbond hydrophobic surface.

Treatment	Gelbond hydrophilic film	Gelbond hydrophobic film	Hydrophobic + 40 μl fruit wax	Hydrophobic + 20 μl paraffin wax	Hydrophobic + 60 μl beeswax
Contact angle (°)	31° ± 0.07	74.63° ± 1.24	101.05° ± 0.11	101.05° ± 0.25	101.05° ± 0.55

### Quantitative RT-PCR (qRT-PCR) Analysis of Gene Expression

Conidia harvested from 5-day-old cultures were washed with sterile distilled water and suspensions were filtered through four layers of cheesecloth to separate hyphal fragments. Conidia suspensions (5 × 105) were placed onto hydrophobic film coated with or without fruit wax for different time periods. Total RNA was extracted from 5 × 105 conidia using TRNzol reagent (QIAGEN, Shanghai, China) according to the manufacturer’s protocol. Reverse transcription was performed using 2 μg of RNA. *GAPDH* was used as an internal control. For quantitative RT-PCR (qRT-PCR) analysis, amplifications were performed using a Bio-Rad CFX96 real-time thermal cycler and QIAGEN QuantiNova SYBR® Green PCR Kit. Relative gene expression was calculated using the 2^−△△ct^ method, as described (Livak and Schmittgen, 2001). The primers shown in Table 2 were used for PCR reactions.

**Table 2:** Primers used in the study.

	Gene	Forward (5′–3′)	Reverse (5′–3′)
Cloning primer	*AaPLC1*	CCATGTCGCTGCTACACGACACCTACT	GAGTGGCGTCATGTTGCAAGCTCACACTAT
	*AaPLC2*	CATGACGCCCACCAAAGACAATAAGCT	TCAAGCCGTACTGACCGTCTTCTTGAT
	*AaPLC3*	CCTACGTGCCACAACTACATTTATTCTAACATG	GGAGAATCGTGTATTGAGACGTTATACACTAG
Quantitative primer	*AaPLC1*	GCCATCGTAGGCGTCAAA	GGTGCCCAGTTCTCGGATAG
	*AaPLC2*	ACAGGTGGCTGGGTTCTCAA	GGTTGTCTTCGTCTTTTGCTTG
	*AaPLC3*	CTCGTCGCACAACACTTACC	TCTCACATACTTGGCGGAAT

### *In vitro* Assays

#### Conidial Germination and Appressorium Formation Assays

Spore suspensions (1 ml) were centrifuged at 5,000 rpm for 5 min, supernatants were discarded, and 1 ml of 10 μM neomycin and 10 μM neomycin + 0.1 mM CaCl_2_ was added to the precipitate. Sterile water was added as a control. The Gelbond film was cut into square sections (5 cm long and 2 cm wide) and coated with 20 μl paraffin, 40 μl fruit wax, and 60 μl beeswax, respectively. Samples were placed onto clean slides to ensure hydrophobicity (contact angle of 101°) (Table 1). Spore suspensions (20 μl) were placed on hydrophobic and hydrophilic Gelbond surfaces coated with or without wax and placed on a humid petri dish. Spore germination and appressorium formation were determined after 2, 4, 6, and 8 h incubation at 28°C through direct microscopic examinations. A minimum of 100 conidia per replicate were assessed (*n* = 3 replicates per treatment). Experiments were repeated on a minimum of three independent occasions.

#### Mycelial Growth of *A. alternata*

After sterilization, neomycin (10 μM) and exogenous CaCl_2_ (0.1 mM) were added to a PDA medium at ~40°C. Spore suspensions (2 μl) were inoculated on the plate and plates were placed at 28°C for incubation. Colony diameters were assessed at 3, 5, and 7 days post-incubation.

#### Mycotoxin Production Assays

Mycotoxin extractions were performed as described by Wang et al. (2016) with minor modifications. Fungi were cultured in PDA at 28°C for 4 days, and ~0.5 g of the samples were filtered and homogenized. Neomycin-treated mycelia were transferred into 10 ml centrifuge tubes, to which 2.5 ml of acetonitrile/water (4:1, v/v) containing 0.3% formic acid was added. Samples were vortexed and mycelia were extracted at 150 rpm for 30 min at room temperature. Subsequently, 0.25 g anhydrous MgSO_4_ and 0.04 g NaCl were added and homogenized for 1 min. Homogenates were centrifuged at 8,000 rpm for 10 min, and supernatants were extracted and filtered through a 0.22 organic membrane, to a volume of 1.2 ml and prepared for high performance liquid chromatography (HPLC) analysis.

TEN, AOH, AME, and ALT were isolated and qualitatively analyzed through mass spectrometry (Agilent 1290, Anjielun, Shenzhen, China) equipped with an electrospray ionization (ESI) source. HPLC conditions were as follows: column, C18 (250 × 4.6 mm, 5 μm); column temperature, 35°C; injection volume, 5 μl; mobile phase A: deionized water, mobile phase B: methanol; gradient elution conditions: A after 70% retention for 1 min, after falling to 50% within 2 min, continued to drop to 10% within 1 min, maintained for 2 min, increased to 90% within 0.1 min, kept for 2 min; tassel 0.005 ml/s; total running time, 7.1 min. The mass spectrometry parameters of four *Alternaria* toxins, including monitored ions, cone energy, and collision energy are shown in Table 3.

**Table 3:** Optimized multiple reaction monitoring (MRM) parameters of the mycotoxins.

	Ionization mode	Parent ion	Qualitative ion	Keep time (min)	Quantitative ion	Fragmentation voltage	Collision energy
Alternariol (AOH)	ESI^−^	257.0	213.0	2.37	147.2	40	32
Alternariol monomethyl ether (AME)	ESI^−^	271.0	256.0	2.85	228.0	32	20
Allenuene (ALT)	ESI^+^	293.1	257.2	3.33	239.1	85	15
Tentoxin (TEN)	ESI^+^	415.2	312.3	3.66	189.0	110	30

### *In vivo* Pathogenicity Assays

The effects of neomycin treatment on the virulence of *A. alternata* were assessed according to previously described methods (Moscoso-Ramírez et al., 2013). Prior to the experiments, fruits were selected, randomized, soaked in 1% sodium hypochlorite solution, and three holes were introduced with stainless steel nails (3 mm wide and 3 mm deep). Different concentrations of inoculum were prepared following previous procedures. Condial suspensions (20 μl) were added and treated fruits were incubated at room temperature (20 ± 2°C) and 90% RH. Lesion diameters were determined after 9 days of storage.

### Statistical Analysis

Data were analyzed using Microsoft Excel 2007 and graphs were plotted using Origin 8.0. All values are representative of the mean ± standard deviation. Data were analyzed using a one-way ANOVA with Duncan’s multiple-range test using SPSS software (version 19.0, SPSS Inc., Chicago, IL, USA). Statistical significance was evaluated at the *p* < 0.05 level.

## Results

### Cloning and Characterization of the PLCs

**Figure 1:**
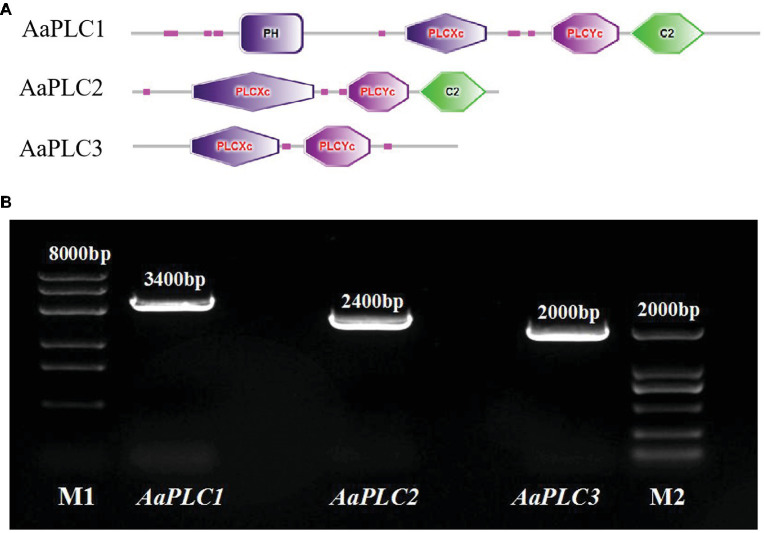
Characteristics of the *Alternaria alternata* phospholipase C (PLC) gene. Schematic of conserved domain predictions of the *AaPLC* gene products **(A)**. PCR amplification of PLC encoding genes in *A. alternata* (primers are shown in Table 2), M1, DL8000 DNA marker; M2, DL2000 DNA marker **(B)**.

*AaPLC*s were cloned from the genome of *A. alternata* (txid5599) and analyzed by bioinformatics. Three putative PLC genes (designated *AaPLC1*, *AaPLC2*, and *AaPLC3*) were identified through a genome database search. *AaPLC1*, *AaPLC2*, and *AaPLC3* were found to contain 3315, 2416, and 2010 bp open reading frames, which encoded proteins of 1116, 669, and 574 amino acids, respectively. The deduced protein sequences of *AaPLC1* contained a pleckstrin homology (PH) domain, X/Y catalytic domains, and a C2 (calcium-binding) domain. The sequences of *AaPLC2* contained X/Y catalytic domains and a C2 domain but no PH domain. Unlike *AaPLC1* and *AaPLC2*, the *AaPLC3* sequence contained only the X/Y catalytic domain (Figure 1).

### PLC Expression Is Enhanced on Hydrophobic and Wax-Extracted Surfaces

**Figure 2:**
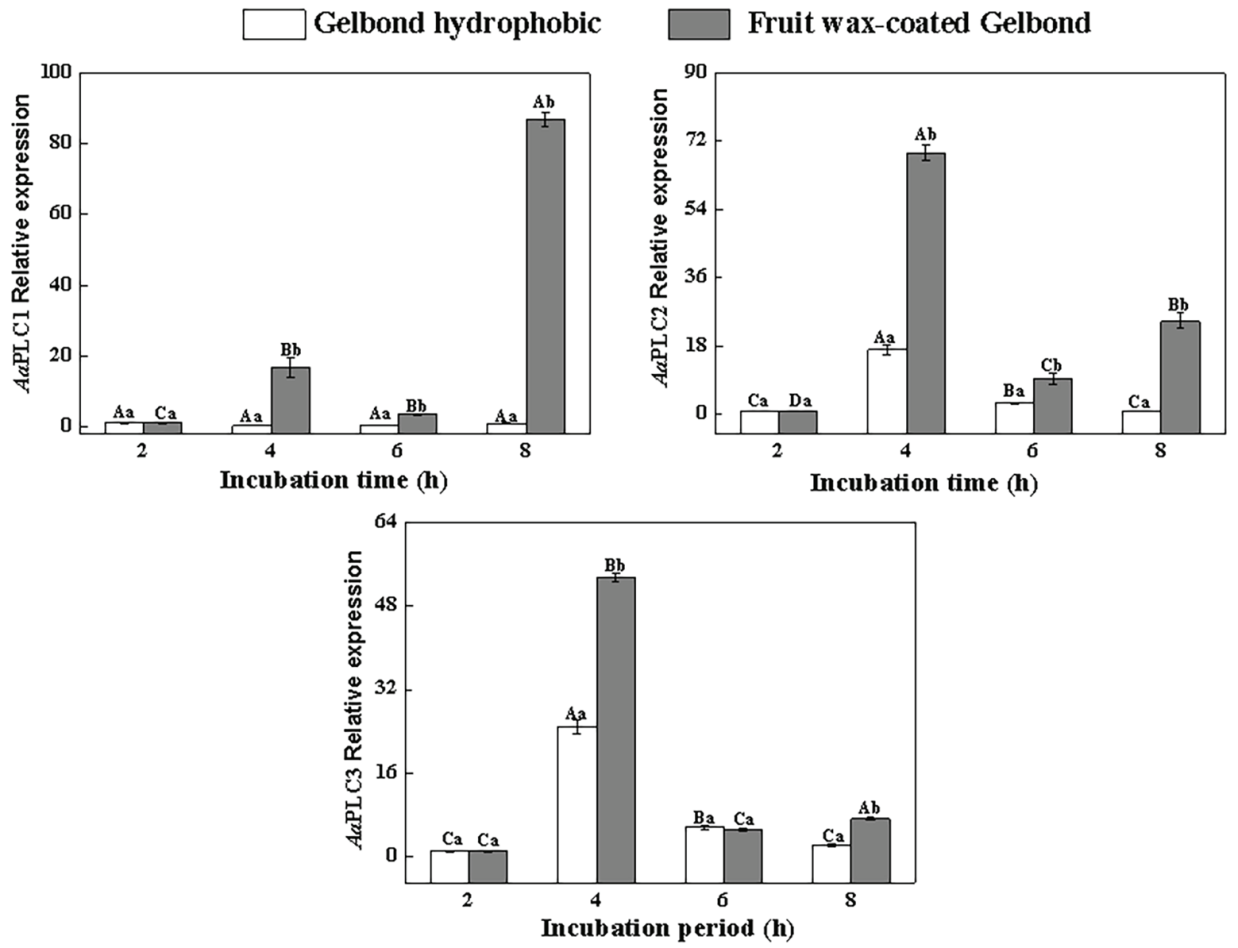
Relative expression levels of *AaPLC1*, *AaPLC2*, and *AaPLC3* during infection. Vertical lines indicate the standard error (±SE) of the means. Uppercase letters indicate inter-group differences. Lowercase letters indicate intra-group differences. Different letters indicate significant differences (*p* < 0.05).

All three *AaPLC*s were significantly upregulated on fruit wax extract-coated surfaces during infection, but the levels of induction were variable (Figure 2). During spore germination (2–4 h), all three *AaPLC* genes were significantly upregulated. Among them, *AaPLC2* and *AaPLC3* of *A. alternata* on the fruit wax surface peaked at 4 h post-incubation and were 69‐ and 53-fold higher than those of the control group. *AaPLC1* was also upregulated 14-fold. The expression of all three genes was significantly downregulated during the germ tube elongation period (4–6 h) and increased during aprressorium formation (6–8 h). In particular, the expression of *AaPLC1* significantly increased on the fruit wax-coated surface, with values 87-fold higher than those measured after 2 h of incubation. The expression of the three *AaPLC* genes of *A. alternata* remained stable under hydrophobic films during conidia development, excluding *AaPLC2* and *AaPLC3*, which were upregulated after 4 h of incubation (Figure 2).

### Inhibition of PLC Reduces Vegetative Growth, Conidia Germination, and Appressorium Formation in *A. alternata* on Hydrophobic and Fruit Wax Extract-Coated Surfaces

#### Concentration Dependent Effects of the PLC Inhibitors on Fungal Infection

**Figure 3:**
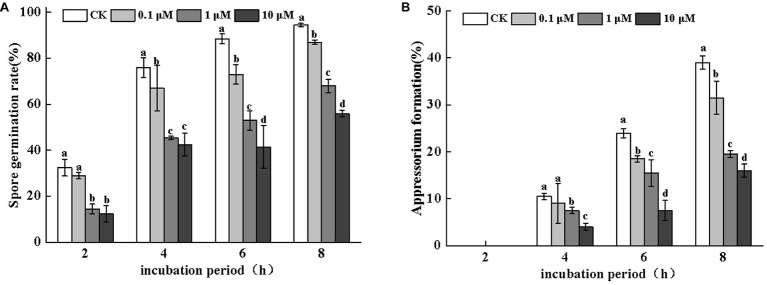
Effects of neomycin on spore germination **(A)** and appressorium formation **(B)** in *A. alternata*. Conidial suspensions treated with a range of concentrations of neomycin were plated onto the hydrophobic surface of Gelbond. Vertical lines indicate standard error (±SE) of the means. Different letters indicate significant differences (*p* < 0.05).

Neomycin treatment led to a loss of conidial germination and appressorium formation of *A. alternata* in a dose-dependent manner. Appressorium formation was more severely inhibited (Figure 3). After 8 h of incubation, the inhibitory effects of neomycin at concentrations of 0.1, 1, and 10 μM on spore germination were 7.9, 28, and 40.7%, respectively (Figure 3A). Appressorium formation in *A. alternata* treated with 10 μM neomycin decreased by ~58.9% (Figure 3B).

#### Effects of Neomycin on the Vegetative Growth of *A. alternata*

**Figure 4:**
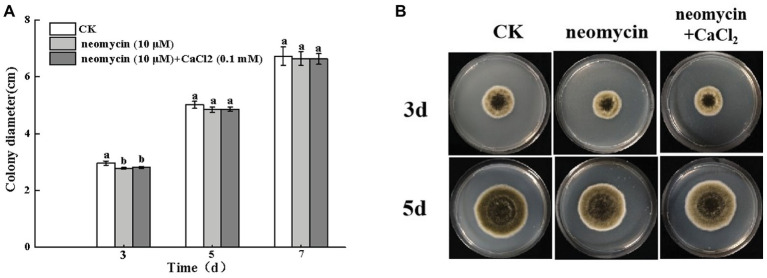
Effects of PLC on *A. alternata* mycelial growth **(A)** and colony morphology **(B)**. Condia (1 × 10^6^) from *A. alternata* were inoculated onto potato dextrose agar (PDA) medium and plates were imaged after 3 and 5 days incubation at 28°C. Vertical lines indicate standard error (±SE) of the means. Different letters indicate significant differences (*p* < 0.05).

The colony diameter of *A. alternata* increased with incubation time. However, no significant differences were observed following neomycin and neomycin + CaCl_2_ treatment in comparison to the control group (Figure 4A). The morphology of *A. alternata* was also unaffected by neomycin and neomycin + CaCl_2_ treatment (Figure 4B).

#### Role of the Hydrophobic Surface in the Induction of Infectious Structures

**Figure 5:**
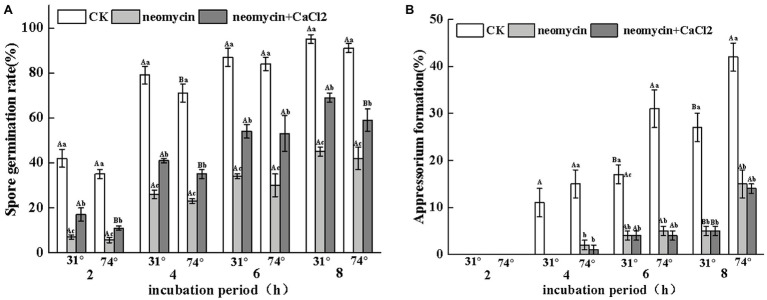
Effects of neomycin on spore germination **(A)** and appressorium formation **(B)** in *A. alternata* induced by hydrophobicity. Conidial suspensions treated with neomycin (10 μM) and exogenous Ca^2+^ (1 mM) were placed onto the hydrophilic and hydrophobic surfaces of Gelbond. Vertical lines indicate standard error (±SE) of the means. Uppercase letters indicate inter-group differences. Lowercase letters indicate intra-group differences. Different letters indicate significant differences (*p* < 0.05).

To evaluate the regulatory role of PLC on the infectious structures of *A. alternata* on hydrophobic surfaces, the rates of spore germination and appressorium formation were determined on hydrophilic and hydrophobic surfaces through the addition of neomycin to the spore suspensions. As shown in Figure 5A, the rates of spore germination of neomycin, neomycin + CaCl2 treated, and non-treated *A. alternata* increased over time. However, no significant differences were observed between hydrophilic and hydrophobic surfaces. Neomycin treatment significantly impaired the spore germination of *A. alternata* (*p* < 0.05), and exogenous CaCl_2_ treatment partially reversed this impairment (Figure 5A). Hydrophobicity with high contact angles significantly induced appressorium formation. After 4 h, the rates of appressorium formation in Gelbond hydrophobic film (74.63° ± 1.24) were 1.4-fold higher than those of hydrophilic film (31° ± 0.07). Neomycin treatment significantly delayed appressorium formation. After 4 h of neomycin treatment, appressorium formation on the hydrophobic surface decreased by 86.6%. Exogenous Ca^2+^ failed to alleviate the decrease in appressorium formation (Figure 5B).

#### Role in Wax Induced Pre-penetration Structures

**Figure 6:**
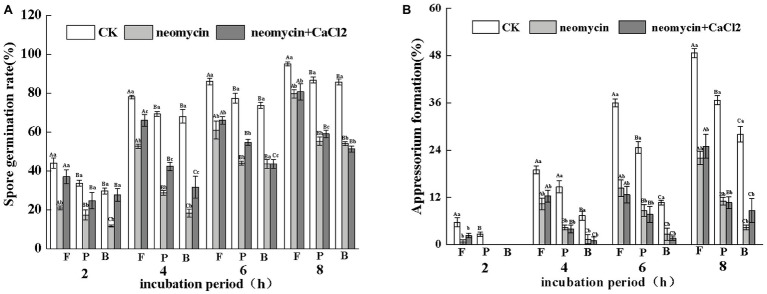
Effects of PLC on spore germination rates **(A)** and appressorium formation rates **(B)** induced by fruit wax (F), paraffin (P), and beeswax (B). Conidial suspensions treated with neomycin (10 μM) and exogenous Ca^2+^ (1 mM) were incubated on inductive hydrophobic membranes coated with different waxes. Vertical lines indicate the standard error (±SE) of the mean. Uppercase letters indicate inter-group differences. Lowercase letters indicate intra-group differences. Different letters indicate significant differences between the treatments (*p* < 0.05).

Under the same hydrophobicity (a contact angle of 101°), fruit wax (F), paraffin (P), and beeswax (B) influenced the spore germination of *A. alternata* to different levels. Fruit wax showed the strongest induction, but no significant differences between paraffin‐ and beeswax-coated surfaces were observed. Neomycin treatment reduced the spore germination induced by different wax-coated surfaces, while exogenous CaCl_2_ partially reversed the decrease. After 4 h of incubation, the spore germination of exogenous CaCl_2_ on fruit wax-coated surfaces was 1.27-fold higher than that of neomycin treatment (Figure 6A). Appressorium formation of *A. alternata* was significantly enhanced under fruit wax (F), followed by paraffin wax (P). Appressorium formation on the fruit wax-coated surface was 1.4‐ and 2.7-fold higher than those of paraffin (P) and beeswax (B) surfaces, respectively. Neomycin treatment on the fruit wax-coated surface significantly reduced appressorium formation rates by 47.4% compared to controls after 4 h of incubation. In contrast, the addition of exogenous CaCl_2_ failed to influence appressorium formation (Figure 6B).

#### PLC Regulates Fungal Pathogenicity

**Figure 7:**
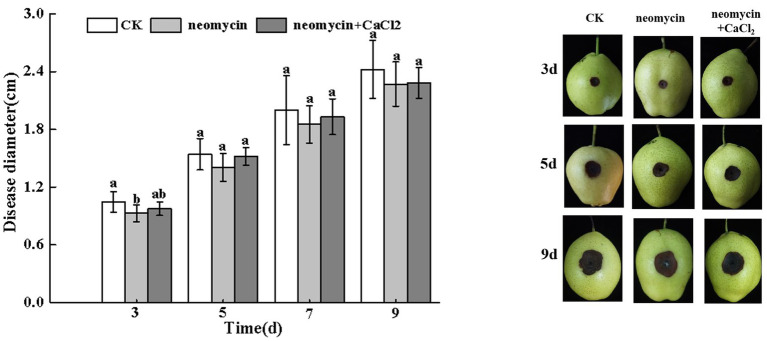
Effects of neomycin and exogenous Ca^2+^ on the black spots of Zaosu pear. Lesions were measured at 3, 5, 7, and 9 days, respectively. Vertical lines indicate standard error (±SE) of the means. Different letters indicate significant differences between treatments according to Duncan’s multiple range test (*p* < 0.05).

As shown in Figure 7, the invasive growth of *A. alternata* in wounded inoculated Zaosu pear was not significantly inhibited by neomycin treatment. The addition of exogenous CaCl_2_ also failed to influence black rot development in pear fruit (Figure 7).

#### PLC Influences Mycotoxin Production in *A. alternata*

**Figure 8:**
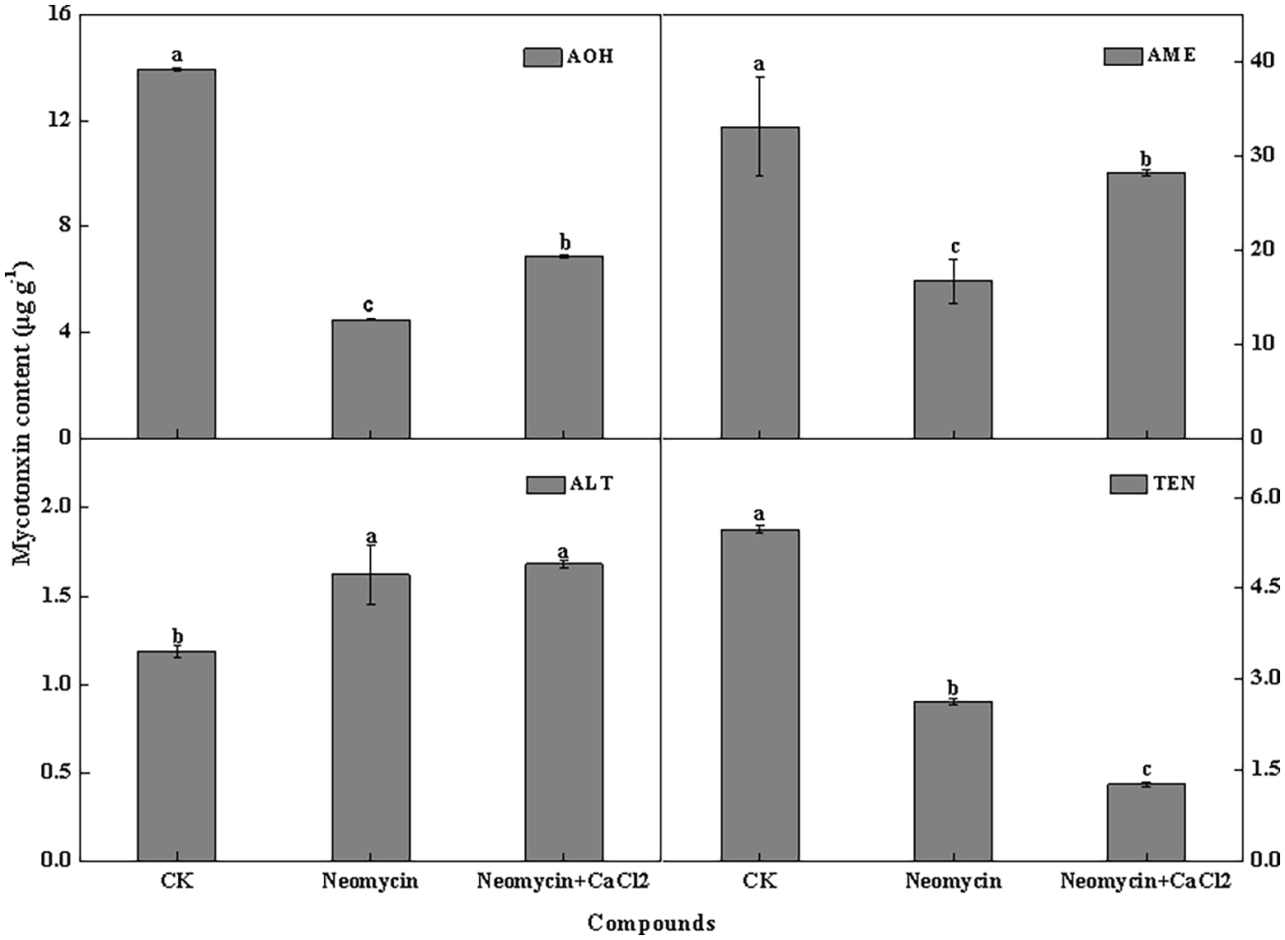
Alternariol monomethyl ether (AME), alternariol (AOH), altenuene (ALT), and tentoxin (TEN) content of *A. alternata* treated with neomycin and exogenous Ca^2+^. Metabolites were assessed in triplicate. Vertical lines indicate the standard error (±SE) of the means. Different letters indicate significant differences between treatments (*p* < 0.05).

PLC inhibitor treatment significantly impacted mycotoxin production in *A. alternata*, but its influence varied among the different mycotoxins. For AOH, AME, and TEN, neomycin treatment led to decreases of 67.7, 49.4, and 52.0%, respectively. Exogenous Ca^2+^ partially reversed the loss of AOH and AME. However, ALT content in neomycin treated *A. alternata* increased by 36.4%, with exogenous Ca^2+^ treatment having no influence on ALT content (Figure 8).

## Discussion

A range of environmental cues and cellular signals that modulate the infectious morphogenesis of plant-pathogenic fungi have been identified. Hardness, hydrophobicity, host surface chemicals, waxes, and ethylene are key determinants of the formation of infection structures (Ahn and Suh, 2007). Appressorium formation of *A. alternata* was induced by hydrophobicity (Figure 5), consistent with previous studies (Lee and Dean 2006 Mendoza et al., 2009) in *Ustilago maydis* and *M. grisea*. Appressoria in *C. graminicola* and other fungi are also induced by the attachment of the germ tube tip to firm hydrophobic surfaces (Chaky et al., 2001). In addition, fruit wax-, paraffin-, and beeswax-coated surfaces of similar hydrophobicity significantly induced spore germination and appressorium formation in *A. alternata*. Appressorium formation on the pear wax-coated surface was 1.4‐ and 2.7-fold higher than paraffin and beeswax surfaces, respectively (Figure 6). These data suggest that specific compounds in pear wax extracts contribute to *A. alternata* infection. Studies have indicated that waxes from different plants show differences in their chemical composition and constituents (Nawrath, 2006). These properties largely dictate host-pathogen recognition and inhibit or stimulate spore germination and infection structure formation. Octacosanol is responsible for the induction of infection structures in wheat stem rust *P. graminis* (Reisige et al., 2006) and barley powdery mildew *B. graminis* (Zabka et al., 2007). However, other studies (Feng et al., 2009) found that long-chain alkanes, as opposed to alcohols, were the most efficient wax component for triggering germination and appressorium development in *B. graminis*. Therefore, the stimulatory functions of plant waxes may vary and are dependent on plant cultivars, individual wax composition, and different host-fungal interaction systems.

All PLC isotypes contain the X and Y domains that form the enzyme catalytic core. Fungal PI-PLCs are similar to the PLC-δ isoforms of mammals (Testerink and Munnik, 2005). Three PLC genes were identified in the *Alternaria* genome, all of which have conserved PLC catalytic domains X and Y. The C2 domain is only present in *AaPLC1* and *AaPLC2* but not in *AaPLC3*. Such variations are conserved in other fungi, including *M. oryzae* (Choi et al., 2011) and *B. cinerea* (Schumacher et al., 2008). *A. alternata* PLCs were cloned and shown to be identical to the PLCs of other fungi (Gavric et al., 2007; Choi et al., 2011). RT-qPCR analysis showed that pear wax extracts enhanced *AaPLC* expression, and that the expression levels of *AaPLC1* peaked at 8 h, while those of *AaPLC2* and *AaPLC3* peaked at 4 h post-incubation (Figure 2). The variation in the degree and stage of three PLCs may be due to their varying regulatory functions. Based on the data presented in this study, we speculate that *AaPLC1* of *A. alternata* may contribute to appressorium formation, while *AaPLC2* and *AaPLC3* contribute to spore germination. The specific regulatory mechanisms now require further evaluation.

Different methods of regulation and the expression of PLCs account for their distinct roles in a variety of cellular processes, including cell development, cell proliferation, and gene expression (Gavric et al., 2007; Choi et al., 2011). Pharmacological experiments showed that treatment with neomycin, an inhibitor of PLC, synchronously reduced conidial germination and appressorium formation in a dose-dependent manner. In particular, appressorium formation was seriously impaired. At concentrations of 10 μM, the rate of appressorium formation decreased by 58.9% after 8 h incubation (Figure 3). However, in *C. miyabeanus*, ~64% of appressorium formation was inhibited by exogenous neomycin treatment at 100 μM (Uhm et al., 2003). Such differences were related to the concentration of neomycin applied and the fungi examined. Neomycin treatment significantly reduced the rates of appressorium formation in *A. alternata* by 47.4% on fruit wax-coated surfaces, suggesting that PLC is indispensable for the infection structures of *A. alternata* in response to fruit surface cues. PLC has also been shown to be required for conidium and appressorium formation and pathogenicity in *B. cinerea* (Schumacher et al., 2008) and in the rice blast pathogen *M. oryzae* (Rho et al., 2009). In addition, in *C. cinerea*, the inhibition of PI-PLC led to decreased conidia and basidiospore germination (Oh et al., 2012). The IP_3_ generated by PLC serves as an intracellular Ca^2+^ channel activator for the maintenance of Ca^2+^ homeostasis, through stimulating its release from intracellular stores in the vacuoles or other organelles (Taylor and Thorn, 2001). Therefore, the treatment of conidia with neomycin blocked the release of intracellular Ca^2+^ ions. Ahn and Suh (2007) investigated the effects of exogenous Ca^2+^ concentrations on infection structure formation of *C. miyabeanus*. The results showed that the addition of CaCl_2_ at 1 mM did not affect germination, but appressorium formation was inhibited to ~51% and nearly abolished at concentrations of 10 mM and 100 mM CaCl_2_, respectively. In this study, we showed that exogenous 0.1 mM CaCl_2_ could partially reverse the impaired spore germination caused by neomycin treatment, but did not affect the destruction of appressorium formation (Figures 5, 6). Therefore, intracellular Ca^2+^ homeostasis is more important than Ca^2+^ influx for appressorium formation in *A. alternata*.

PLC-mediated Ca^2+^ signaling occurs in response to phytopathogen pathogenicity. Onion epidermal cells were used to study PLC1 mutants of *M. oryzae* penetration, in which *Moplc1* conidia infrequently formed appressoria and failed to penetrate plant cells, As such, no invasive hyphae were formed and the pathogenicity of the fungus was compromised (Rho et al., 2009). The lack of corresponding PLC activity of *Fusarium graminearum* leads to the reduction of mycelial growth and pathogenicity (Zhu et al., 2015). Pathogenicity defects of plant fungi were not entirely attributed to the marked reduction in appressorium formation, which may also be related to the mode of invasive growth or penetration. However, this study showed that neomycin treatment did not significantly inhibit the invasive growth of *A. alternata* in pear fruit following wound inoculation (Figure 7), suggesting that PLC is required for fruit surface recognition and the pre-penetration regulation of *A. alternata*. Neomycin treatment also reduced the AOH, AME, and TEN content but modestly increased ALT (Figure 8). Similar studies reported that PLC inhibition leads to a dose-dependent decrease in cercosporin biosynthesis in *Cercospora nicotianae* (Chung, 2003). In *F. graminearum*, *Fgplc1* mutants led to a reduction in DON production and virulence during infection in flowering wheat heads (Zhu et al., 2016). These results confirm that PLC in the different fungal species contributes to the regulation of secondary metabolism. However, genetic methods are required to further unravel the specific mechanisms of this effect.

## Conclusions

In conclusion, fruit wax extracts significantly upregulate the expression of the three PLC genes identified in *A. alternata*. Both hydrophobic surfaces and wax extracts of pear led to the stimulation of spore germination and appressorium formation in *A. alternata*. Neomycin, an inhibitor of PLC, impaired the induction of *A. alternata* spore germination and appressorium formation. Neomycin also affected mycotoxin production in *A. alternata*. These results indicate that PLC-mediated Ca^2+^ signaling is required for the infectious structures of *A. alternata* during recognition and the response to physicochemical signals at the pear surface. The molecular mechanisms of PLC regulating *A. alternata* growth, sporulation, secondary metabolism, and pathogenicity now require further elucidation.

## Data Availability Statement

The raw data supporting the conclusions of this article will be made available by the authors, without undue reservation, to any qualified researcher.

## Author Contributions

YLc, YB, YH, and DP conceived and designed the experiments. YH, DL, YD, TW, MZ, XZ, and YLx performed the experiments. YH and DL analyzed the data. YH and YLc prepared the manuscript. All authors have read and agreed to the published version of the manuscript.

## Conflict of Interest

The authors declare that the research was conducted in the absence of any commercial or financial relationships that could be construed as a potential conflict of interest.
